# Gain Saturation of Encapsulated CdTe-Ag Quantum Dot Composite in SiO_2_

**DOI:** 10.3390/nano14231950

**Published:** 2024-12-04

**Authors:** Minwoo Kim, Agna Antony, Inhong Kim, Minju Kim, Kwangseuk Kyhm

**Affiliations:** Department of Optics & Cogno-Mechatronics Engineering, Pusan National University, Busan 46241, Republic of Korea

**Keywords:** quantum dot composite, optical gain, logistic curve, gain saturation, plasmonic effect

## Abstract

Amplified spontaneous emission of CdTe and CdTe-Ag quantum dot composites were compared for increasing the optical stripe length, whereby optical gain coefficients for various emission wavelengths were obtained. In the case of CdTe-Ag nanoparticle composites, we observed that plasmonic coupling causes both optical enhancement and quenching at different wavelengths, where the amplified spontaneous emission intensity becomes enhanced at short wavelengths but suppressed at long wavelengths (>600 nm). To analyze the logistic stripe length dependence of amplified spontaneous emission intensity, we used a differential method to obtain the gain coefficient beyond the amplification range. This analysis enabled us to find the limit of the commonly used fitting method in terms of a threshold length and a saturation length, where amplification begins and saturation ends, respectively.

## 1. Introduction

Optical gain in semiconductor nanomaterials plays a major role in the design of optoelectronic devices such as lasers and optical amplifiers. It measures how effectively a medium amplifies photons through stimulated emission, and gain coefficient is a crucial parameter for determining the lasing capabilities of a material. By examining optical gain, we can investigate carrier dynamics and light–matter interaction in these materials [1,2,3]. Research on optical gain in semiconductor nanomaterials has a long history, and numerous studies continue to be conducted worldwide. Among the various nanomaterials, quantum dots (QDs) have been gained a lot of attention due to their size-dependent properties.

A lot of attention is drawn to quantum dots as a gain medium due to their strong confinement and tunability [4,5]. The application of CdSe quantum dots to a laser diode was addressed, and the presence of optical gain in QD-LED was demonstrated based on high current injection [6]. Currently, CdTe QDs are also captivating interest for their high quantum yield and narrow direct bandgap (1.44 eV). Compared to CdSe and CdS QDs, CdTe QDs are actively utilized with integrated nanocomposite structures in aqueous media [7], where the bottom-up properties are applied to nonlinear optics, magnetic agents, and catalysis as functional materials. CdTe QD-based thin film technology is also used in photovoltaic (PV) applications [8]. Moreover, their exceptional fluorescence, broad optical absorption tunability, chemical stability, and ease of biomolecule conjugation also make them excellent alternatives to traditional fluorescent dyes for biological applications [9,10]. The composition-dependent tunability and excellent photochemical stability of CdTe QDs further enhance their performance. For example, combining CdTe QDs with other materials can improve photocatalytic efficiency by boosting adsorption capacity and stability. Additionally, CdTe QDs are extensively studied in medical imaging due to their temperature-dependent luminescence, where emission line shifts correlated with temperature variations can be used for thermal sensing [11]. Their use in biomedical fluorescent labeling, including two-photon excitation with long wavelengths, further expands their applications in advanced diagnostics and therapeutics [12].

On the other hand, metal QDs with discrete, size-tunable electronic transitions exhibit strong local surface plasmon resonance (LSPR), where free electrons resonate with incident light, generating intense localized electromagnetic fields. Provided that the LSPR is coupled with the exciton in semiconductor QDs, the exciton oscillator strength becomes enhanced significantly as a consequence of the Purcell effect [13,14,15]. Therefore, a hybrid structure of metal–semiconductor QD composites present exciting opportunities, where the size-dependent optical and electronic properties of emitting QDs can be combined with the SPR of metal QDs. The development of CdTe nanowires using Au as a catalyst has been studied and documented before [16].

The enhancement and quenching of photoluminescence (PL) in CdTe-Ag QD composites have been previously reported, but they depend on several factors such as the resonance tuning between the absorption of CdTe QDs and the SP energy of Ag QDs, the geometry, and the separation distance [17,18]. Similar plasmonic enhancement was observed in copper-doped CdSe nanoplatelets. In this case, the magneto-optical properties were found to be highly dependent on their structural parameters [19]. However, the plasmonic effect of CdTe-Ag QD composites was rarely considered as a gain medium. Recently, the presence of optical gain in CdSe/CdS quantum wells was reported. Although the edge emission was measured using a capillary waveguide, the length dependence of the amplified spontaneous emission was not considered so that practical model gain was not obtained [20].

In this study, we utilize the variable stripe length method (VSLM), which was developed by Shaklee and Leheny in the 1970s, to investigate the optical gain properties of the material. The VSLM is used to measure the modal gain along an optical stripe rather than the transient intrinsic gain of materials. Compared to the transient absorption technique using femtosecond pulses, the VSLM enables us to measure the modal optical gain using a simple and convenient setup [21,22]. In the VSLM setup, the sample is optically pumped with a stripe-shaped beam, and the intensity of the edge emission is measured as a function of the stripe length. The presence of gain results in an exponential increase in edge emission intensity for an increasing stripe length, while spontaneous emission leads to a linear increase with stripe length [23,24,25]. For the gain sample, CdTe QDs and CdTe-Ag QD composites were utilized, which are encapsulated in SiO_2_ at 1:1 and 1:10 ratios. We measured the edge emission spectra for all samples at 4 K, where spectral broadening and wavelength-dependent quenching and enhancement of the edge spectrum were observed. Additionally, the gain spectra of the samples were compared using two different techniques, the differential method and the fitting method. Furthermore, the limit of the fitting method and advantages of the differential method were considered regarding gain saturation.

## 2. Materials and Methods

**Figure 1:**
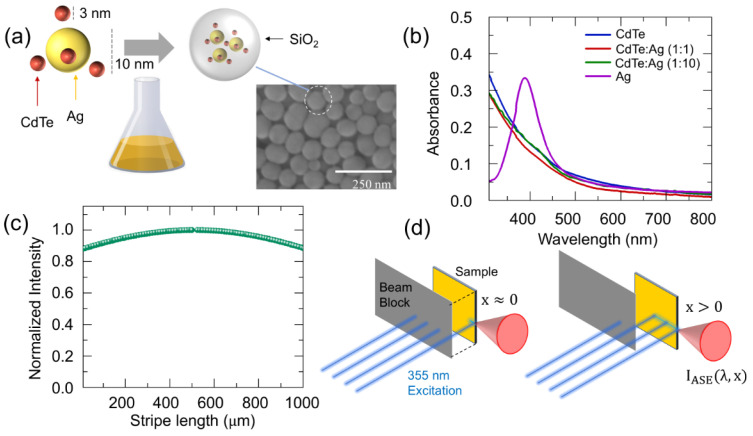
(**a**) CdTe-Ag QD composites encapsulated with SiO_2_ are shown schematically with a Scanning Electron Microscopy (SEM) image. (**b**) The absorption spectrum of CdTe QDs, Ag QDs, and QD composites with 1:1 (CdTe:Ag) and 1:10 ratios. (**c**) The intensity distribution of an optical stripe. (**d**) The experimental setup of the VSLM for x≈0 and x>0.

As shown in Figure 1a schematically, Ag QDs with an average diameter of ~10 nm were synthesized using a modified chemical reduction method based on [26]. Detailed synthesis procedures and conditions are provided in the Supporting Information (Section S1). For CdTe QDs with an average diameter of ~3 nm, a commercial product from Sigma-Aldrich (St. Louis, MO, USA) was used. To study the plasmonic enhancement effect on the optical properties of CdTe QDs, CdTe QDs were mixed with Ag QDs in different molar ratios. Three different mixtures were prepared (a pure CdTe QD solution and CdTe QD solutions mixed with Ag QDs at molar ratios of 1:1 and 1:10). To encapsulate CdTe QDs and Ag QDs in a silica (SiO_2_) sphere, the Stöber method was employed. This encapsulation process not only provides structural stability but also significantly enhances the environmental stability of the QDs by preventing oxidation and surface degradation. Detailed encapsulation procedures, including the reaction conditions, are described in the Supporting Information (Section S1) [8]. In brief, the mixtures of CdTe QDs and Ag QDs were added dropwise to a TEOS solution in ethanol under basic conditions (ammonium hydroxide). This process facilitated the hydrolysis and condensation of silica, forming uniform SiO_2_ shells around the CdTe QDs and Ag QDs. As shown in the SEM image inset in Figure 1a, the encapsulated particles had a uniform shell size of ~90 nm, which was observed using a Hitachi S-4800 FESEM at an acceleration voltage of 15 kV and a magnification of 50,000×. The encapsulated particles were collected by centrifugation and washed three times with water and ethanol to remove any unreacted TEOS and byproducts. Previous studies have demonstrated that multiple nanoparticles can be encapsulated within a single SiO_2_ shell at higher particle concentrations [26,27].

Figure 1b shows the absorbance spectrum of the synthesized samples using a SpectraMax M5 UV/VIS spectrometer over the wavelength range from 300 nm to 800 nm. The absorption spectrum of the Ag QDs dispersed in water displays a characteristic localized surface plasmon resonance (LSPR) peak around 389 nm, which corresponds to ~10 nm diameter Ag QDs [26]. On the other hand, the absorption spectrum of the CdTe QDs solution shows a subtle shoulder around 425 nm, which is indicative of the lowest confinement states of an electron–hole pair with a diameter of 3 nm [28]. To clarify the subtle shoulder near 425 nm, the absorbance spectra were replotted on a logarithmic scale (Supporting Information, Section S2), whereby the absorption shoulder becomes distinct and the spectral overlap with that of Ag QDs was also confirmed.

When the sample is excited along an optical stripe, edge emission can be measured [29]. In the presence of optical gain, the edge emission intensity shows an exponential growth for an increasing optical stripe length. A cylindrical lens can be used to prepare an optical stripe, whereby the circular laser beams become a narrow stripe. However, the optical stripe still contains a non-uniform intensity distribution due to the Gaussian intensity distribution of the laser beam. The non-uniformity of the optical stripe can be suppressed by a beam expander. In Figure 1c, we measured the intensity of the optical stripe, where the non-uniformity became suppressed to be ~10% in a stripe length with a range of 1000 μm, and we found that 10% non-uniformity of excitation light is negligible in estimating optical gain.

Figure 1d depicts the VSLM setup used for optical gain measurements. In this setup, a nanosecond pulsed laser operating at a wavelength of 355 nm is used to excite the sample, forming an optical stripe on the sample surface using a cylindrical lens. The length of this stripe is varied by adjusting a movable beam block, allowing precise control of the stripe length (x) [21,22]. As light propagates along the excited stripe, the emission becomes amplified, leading to amplified spontaneous emission (ASE) at the edge of the sample. Even if the beam block sets *x* = 0, diffraction effects cause edge emission signals. This makes it challenging to precisely define the exact location of *x* = 0. Despite this limitation, the edge emission intensity measured across varying stripe lengths still offers valuable insights into the optical gain characteristics of the sample and enables us to assess the enhancement effects of plasmonic QDs on the optical properties of CdTe QDs.

The edge emission intensity, which depends on both the stripe length and emission wavelength, can be quantitatively analyzed to determine the optical gain of the sample. The relationship between the edge emission intensity $I\left(\lambda ,x\right)$, stripe length (x), and emission wavelength (λ) is given by the following differential equation [30]:

(1) $$\frac{dI\left(\lambda ,x\right)}{dx}=g\left(\lambda ,x\right)I\left(\lambda ,x\right)+J_{spon}(\lambda )\Omega$$

where $g\left(\lambda \right)$ represents the modal gain coefficient at a specific emission wavelength (λ), and $J_{spon}(\lambda )\Omega$ is the spontaneous emission density. The term Ω is the solid angle over which the edge emission is collected. Assuming that $g\left(\lambda ,x\right)$ remains constant for the stripe length *x*, the solution to this equation is [24,30,31]

(2) $$I\left(\lambda ,x\right)=\frac{J_{spon}\left(\lambda \right)\Omega }{g\left(\lambda \right)}(e^{g\left(\lambda \right)x}-1)$$

where the edge emission intensity exhibits exponential growth with an increasing stripe length. By fitting the observed intensity $I\left(\lambda ,x\right)$ to this equation, $g\left(\lambda \right)$ can be determined. It is noticeable that $g\left(\lambda \right)$ is a modal gain, which depends on the intrinsic gain of the material $g_{i}$, mode confinement Γ, and optical losses α, as expressed by the following relationship [24,30,31]:

(3) $$g=\Gamma g_{i}-\alpha$$

This analysis allows us to precisely calculate the optical gain and understand how plasmonic QDs enhance the optical properties of CdTe QDs, providing key insights into the amplification efficiency and the effectiveness of plasmonic enhancement.

## 3. Results and Discussion

**Figure 2:**
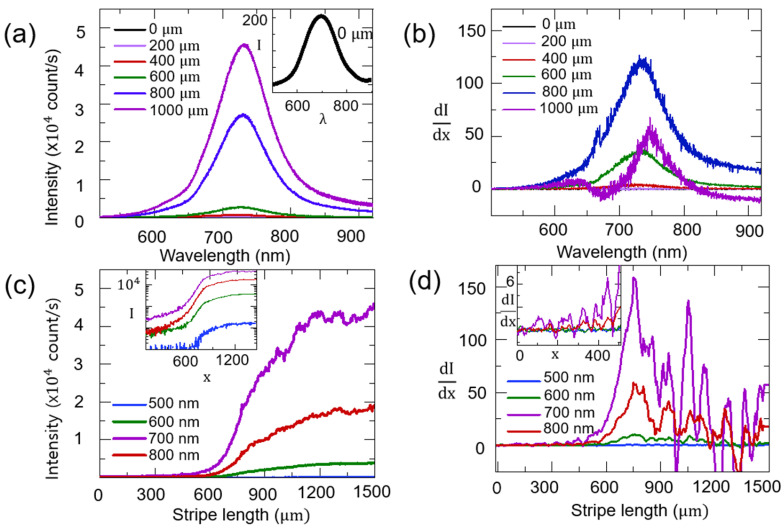
(**a**) Edge emission spectrum of CdTe QDs for increasing stripe lengths at 4 K. Inset shows edge emission at 0 μm stripe length. (**b**) dI(λ,x)/dx spectrum at different stripe lengths. (**c**) Stripe length-dependent edge emission intensity at various wavelengths. Inset shows edge emission intensity in log scale. (**d**) Stripe length dependence of dI(λ,x)/dx at different wavelengths. Short stripe length range (*x* < $x_{th}$) is shown in inset.

Figure 2a shows the edge emission spectrum of CdTe QDs for an increasing stripe length at 4 K. As evidence of amplification, the intensity of the spectrum grows with an increasing stripe length. All curves exhibit a peak at around 720 nm. As the optical stripe length increases to 1000 μm, the edge emission intensity changes significantly in the range between 600 nm and 900 nm. As shown in the inset of Figure 2a, it is noticeable that edge emission is still observed at 0 μm as a result of the laser diffraction by the edge of the beam block. The tail of the edge emission spectrum beyond 900 nm is weak. This can be attributed to the emission from trap states caused by surface defects or the incomplete ligand passivation of the CdTe QD.

In Figure 2b, the derivative spectrum of edge emission dI(λ,x)/dx for an increasing stripe length is displayed. This spectrum is essential for analyzing amplification suppression and gain saturation as a function of the stripe length. For an increasing stripe length up to 800 μm, the spectrum intensity of dI(λ,x)/dx increases over the whole wavelengths due to amplification. However, when a stripe length becomes longer than a saturation length ($x_{sat}\sim \;800\;\mu m)$, the derivative spectrum intensity decreases. For example, at a 1000 μm stripe length, even a negative value of dI(λ,x)/dx is seen near the wavelength of 680 nm and the long wavelengths (>830 nm). This negative derivative implies that optical loss is larger than amplification, which indicates that gain saturation becomes significant at long stripe lengths $\left(>x_{sat}\right)$. Therefore, dI(λ,x)/dx is useful to identify the stripe length range of gain saturation.

In Figure 2c, the stripe length dependence of the edge emission intensity I(λ,x) at various wavelengths was obtained from Figure 2a. Between 400 μm and 800 μm, most of I(λ,x) at various stripe lengths show an exponential increase, as predicted in Equation (2). This can be considered a region of ideal optical amplification without gain saturation. However, the slope dI(λ,x)/dx decreases gradually for x>800 μm. For example, regarding the I(λ,x) value selected at the wavelengths of 700 nm and 800 nm, Equation (2) is no longer valid, and the gain coefficient depends on the stripe length $g\left(\lambda ,x\right)$ for long stripe lengths $\left(>x_{sat}\;\sim \;800\;\mu m\right)$. In addition, I(λ,x) shows a linear dependence with short stripe lengths (<400 μm). These results suggest that the optical stripe length should be longer than a threshold length (${x>x}_{th}$) for amplification, and this is possibly associated with the finite width of the optical stripe (~100 μm), where the gain is affected by the waveguiding effect. In the inset of Figure 2c, *I*(λ,*x*) is shown in a logarithmic scale to clarify the characteristic regions of short $\left(x<x_{th}\right)$, amplification $\left(x_{th}<x<x_{sat}\right)$, and saturation ($x<x_{sat}$) stripe lengths. Although the three regions depend on the emission wavelength, the presence of $x_{th}$ and $x_{sat}$ is evident. It is noticeable that the region of ideal optical amplification is very limited, where Equation (2) is applicable.

To overcome this issue, we utilized an alternative approach known as the differential method. This method directly calculates modal gain from Equation (1) [30,32]:

(4) $$g\left(\lambda ,x\right)=\frac{\frac{dI(\lambda ,x)}{dx}-J_{Spon}(\lambda )\Omega }{I\left(\lambda ,\;x\right)},$$

where the spontaneous term $J_{spon}(\lambda )\Omega$, which consists of the spontaneous emission density $J_{spon}(\lambda )$ and solid angle Ω, can be obtained from the average slope in the short stripe length $\left(x<x_{th}\right)$, where the edge emission intensity shows a linear dependence for stripe length $I(\lambda ,x)\sim (J_{spon}(\lambda )\Omega )x$. As Equation (4) uses the three separate terms, I(λ, x), dI(λ,x)/dx, and $J_{spon}(\lambda )\Omega$, the gain coefficient can be calculated. While the fitting method is applicable only to the amplification region $\left(x_{th}<x<{\;x}_{sat}\right)$, this approach is not limited by the fitting range. Therefore, Equation (4) provides a gain spectrum across the full range of stripe lengths in a straightforward manner [33,34].

Figure 2d shows the stripe length-dependent dI(λ,x)/dx value at different wavelengths. At a 700 nm wavelength, an increase in the slope of dI(λ,x)/dx can be seen in the amplification range $\left(x_{th}∼\;400\;\mu m<x<x_{sat}\;∼\;800\;\mu m\right)$, which indicates the presence of gain. Beyond the saturation length $\left(x>x_{sat}\right)$, a decrease in slope is significant because of gain saturation. The similar feature of dI(λ,x)/dx can also be seen at other wavelengths with a decreased slope. Compared to Figure 2c, this analysis is useful to distinguish the three characteristic regions of stripe lengths. While the logistic curve in Figure 2c is helpful to indicate these regions, there is some ambiguity in defining the exact stripe lengths ($x_{th}$ and${\;x}_{sat}$). This can be clarified with Figure 2d; the point where the constant dI(λ,x)/dx (linear region) starts to change indicates the threshold stripe length ($x_{th}$). The positive slope value of dI(λ,x)/dx denotes the amplification region $\left(x_{th}<x<x_{sat}\right)$, and the point where the slope of dI(λ)/dx becomes negative defines the saturation stripe length ($x_{sat}$). The inset shows dI(λ,x)/dx in the region of short stripe lengths $({x<x}_{th}$), where only spontaneous emission term gives rise to $I(\lambda ,x)\sim (J_{spon}(\lambda )\Omega )x$. Because the spontaneous emission density $J_{spon}(\lambda )\Omega$ is as small as a noise level, dI(λ,x)/dx shows a variation around the expected constant. Interestingly, a periodic feature is also seen in the case of 700 nm and 800 nm. The similar feature was also observed in the saturation region $\left(x>x_{sat}\right)$ as shown in Figure 2d. We found that the dominant period of the stripe length is comparable to the stripe width (~ 100 μm). Therefore, this can be attributed to the waveguide modes in an optical stripe.

**Figure 3:**
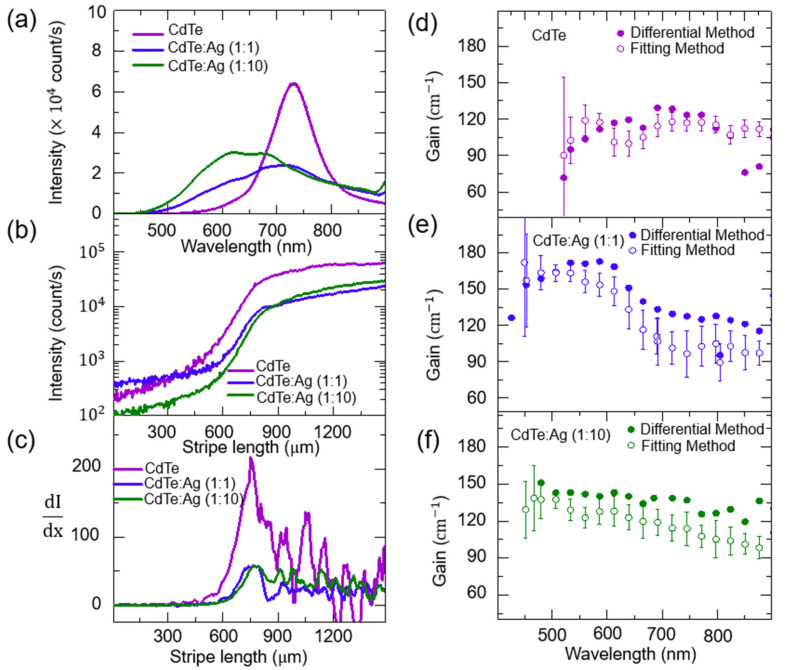
(**a**) Edge emission spectrum of CdTe QDs and CdTe-Ag QD composites at 1:1 and 1:10 ratios. (**b**) Stripe length dependence of edge emission intensity (in log scale) selected at wavelength of 730 nm for CdTe QDs and CdTe-Ag QD composites at 1:1 and 1:10 ratios. (**c**) Variation of dI(λ,x)/dx with stripe length for CdTe QDs and CdTe-Ag QD composites at 1:1 and 1:10 ratios at wavelength of 730 nm. (**d**–**f**) Gain spectrum of CdTe QDs and CdTe-Ag QD composite at 1:1 and 1:10 ratios obtained using fitting method shown in Equation (2) and differential method shown in Equation (3).

As shown in Figure 3a, the edge emission spectra of CdTe QDs mixed with Ag QDs at different ratios exhibit three key trends compared with that of pure CdTe QDs. First, the edge emission spectrum of CdTe-Ag QD composites becomes broadened. The extended spectral bandwidth indicates the presence of plasmonic interactions between CdTe QDs and Ag QDs, whereby more states become involved in the emission process. Second, the dominant edge emission shifts to short wavelengths (<660 nm), which can be attributed to the plasmonic enhancement of the oscillator strength. This observation is consistent with previous studies demonstrating that plasmonic coupling can modulate the energy levels of quantum dots and induce spectral shifts depending on the resonance overlap and interparticle distance [35,36,37]. Regarding the energy conversion from light to the carriers of QDs, it is noticeable that a part of light excitation is lost by the absorption of Ag QDs. Suppose the plasmonic wavelength of Ag QDs is too far to be coupled with the excitons of CdTe QDs, the effective excitation intensity to CdTe QDs decreases for an increased concentration of Ag QDs, resulting in a decrease in PL. However, as the exciton energy of CdTe QDs becomes near the plasmonic energy of Ag QDs, the strong local electric field of the photo-excited Ag QDs enhances the exciton oscillator strength of CdTe QDs. Therefore, the optimum condition of PL enhancement requires both the size of CdTe QDs and the concentration ratio between CdTe and Au QDs. Therefore, only the spectrum range of CdTe QDs from 450 nm to 650 nm is enhanced by the local field of Ag QDs, and the overall suppression of PL intensity from 700 nm to 800 nm can be attributed to the absorption loss of Ag QDs rather than carrier trapping at a short distance. 

In Figure 3b, the stripe length dependence of the edge emission intensity selected at the same wavelength of 730 nm was shown for the three samples of bare CdTe QDs and two kinds of CdTe-Ag QD composites (1:1 and 1:10), where the three characteristic regions of short $(x<{\;x}_{th}),$ amplification $\left(x_{th}<x<x_{sat}\right)$, and saturation $\left(x>x_{sat}\right)$ stripe lengths were evident. These regions are clearly seen, where the stripe length dependence of the edge emission intensity changes from linear to exponential growth near the threshold length $\left(x>{\;x}_{th}\right)$, and the exponential growth begins to deteriorate near the gain saturation region $\left(x>x_{sat}\right)$. As a result, the fitting method is applicable to the limited range $\left(x_{th}<x<{\;x}_{sat}\right)$. However, the differential method is free from the limit, and it provides the stripe length-dependent gain coefficient over the whole stripe length.

Given the stripe length dependence of the edge emission intensity at a certain wavelength I(λ,x) (Figure 3b), it is difficult to find the ideal length range of exponential growth from the logistic experiment result. While the stripe length dependence changes from linear ~$J_{spon}(\lambda )\Omega (x)$ to exponential ~$e^{gx}$ at $x_{th}$, the exponential dependence becomes suppressed near $x_{sat}$. However, the ambiguity in determining $x_{th}$ and $x_{sat}$ is unavoidable. Therefore, dI(λ,x)/dx is useful to clarify the two critical transition lengths of $x_{th}$ and $x_{sat}$. Figure 3c presents the length derivative of the edge emission intensity for the stripe length. dI(λ,x)/dx remains constant with ~$10^{0}$ count/μm for a short stripe length $\left(x<x_{th}\right)$, and a notable increase is seen beyond $x_{th}$ due to the exponential growth of the edge emission intensity (~$e^{gx}$). $x_{sat}$ can be identified by the point where the maximum dI(λ,x)/dx begins to decline abruptly.

Compared with the stripe length-dependent edge emission of CdTe QDs selected at the dominant wavelength of 730 nm $I_{\lambda }(x)$, $x_{th}$ of the edge emission at 730 nm increases as the concentration of Ag QDs increases. The increase in $x_{th}$ is associated with the suppression of the edge emission intensity, and this can be attributed to the absorption loss by Ag QDs. As a result, amplification needs longer stripe lengths, and the amplification range $\left(L_{amp}=x_{sat}-x_{th}\right)$ also shortens; $L_{amp}=381,\;319,\;284\;\mu m$ for CdTe QDs and CdTe-Ag QD composites with ratios of 1:1 and 1:10. In contrast, at 610 nm, $L_{amp}$ increases with the increased concentration of Ag QDs; $L_{amp}=174,\;261,\;290\;\mu m$ for CdTe QDs and CdTe-Ag QD composites with ratios of 1:1 and 1:10, and this can be attributed to the size dependence of plasmonic enhancement, i.e., while optical quenching occurs in the large (>700 nm) CdTe QDs, plasmonic enhancement is induced in the small (<700 nm) CdTe QDs. The detailed wavelength-dependent stripe length analysis, including $L_{amp}$ trends, is also provided in the Supporting Information (Figure S4).

In Figure 3d, the optical gain spectra of pure CdTe QDs obtained by the two different methods were compared. In the case of the fitting method, the gain was obtained from the amplification range of $L_{amp}$, and it is similar to the gain obtained by the differential method at $x_{th}$, which corresponds to the upper limit which corresponds to $x_{sat}$. The large error at short wavelengths can be attributed to a decrease in$\;L_{amp}$. For short wavelengths, we found that $x_{th}$ increases while $x_{sat}$ remains constant. While the edge emission spectrum shows a Gaussian distribution centered at 730 nm, it is noticeable that the gain coefficients show a nearly uniform distribution with 110 ± 20 cm^−1^ over the whole spectrum range (500 nm ~ 900 nm). This result provides us with an important lesson in contrast to intuitive prediction. The gain spectrum does not follow the spectrum shape of edge emission. For example, a large edge emission intensity does not guarantee a large gain coefficient, but a large gain can be induced from small edge emission intensity. The gain coefficient is extracted from the length dependence of the edge emission, but it depends on the three characteristic regions of short (x < $x_{th}$), amplification ($x_{th}$< $x<x_{sat}$), and saturation (x >${\;x}_{sat}$) stripe lengths, and the three regions also depend on the wavelength.

In the case of the CdTe-Ag QD composite with a 1:1 ratio (Figure 3e), we found that the gain spectrum became extended to 400 nm, and the gain coefficients were also enhanced by up to 180 cm^−1^. For short wavelengths below 400 nm, the edge emission intensity is too small to extract a gain coefficient. This spectrum range of gain enhancement may resonate with plasmonic coupling, and we also found that the gain enhancement decreases gradually as the wavelength increases. On the other hand, with a 1:10 ratio for the CdTe-Ag QD composite (Figure 3f), a uniform gain spectrum was obtained, where the gain coefficient also became enhanced compared to that of pure CdTe QDs, and an increase in gain coefficients at short wavelengths (400 nm ~ 600 nm) is significant. Interestingly, we found that the CdTe-Ag QD composite with a 1:10 ratio shows a similar $L_{amp}$ value over the whole spectrum, while pure CdTe QDs show a different $L_{amp}$ value for the wavelength. This implies that the plasmonic effect leads to shortening of the $L_{amp}$ rather than an increase in the intrinsic gain coefficient.

## 4. Conclusions

Plasmonic enhancement was studied in terms of optical gain using a CdTe-Ag QD composite encapsulated in SiO_2_. To obtain the gain spectrum using the variable stripe length method, the differential method was utilized, whereby the limits and pitfalls of the well-known fitting method were addressed in terms of the threshold length and saturation length, and the stripe length dependence of the gain saturation was also revealed. With increased concentration ratio of the Ag QDs to CdTe QDs, we found that the edge emission is both enhanced and suppressed at different spectrum ranges. While plasmonic enhancement appears at relatively short wavelengths (<650 nm), emission suppression occurs at long wavelengths (>650 nm) due to the absorption loss by increased Ag QDs. The same spectral features were confirmed in the gain spectrum.

## Data Availability

All data generated or analyzed during this study are included in this published article.

## Supplementary Materials

The following supporting information can be downloaded at: https://www.mdpi.com/article/10.3390/nano14231950/s1, Figure S1. Stepwise synthesis process of CdTe/Ag QD composites encapsulated in SiO_2_; Figure S2. Absorption spectra of CdTe QDs, Ag QDs, and CdTe-Ag composites at molar ratios of 1:1 and 1:10. The absorption peak of CdTe QDs at ~425 nm and the localized surface plasmon resonance (LSPR) peak of Ag QDs at ~389 nm are visible. The composite spectra show slight shifts and broadenings, which are attributed to plasmonic interactions between CdTe QDs and Ag QDs. Data were measured in solution after particle recovery, assuming consistent concentration across all samples; Figure S3. Integrated ASE spectra of CdTe QDs and CdTe-Ag composites with different Ag QD molar ratios (1:1 and 1:10). The areas under the curves represent the relative ASE intensities, which were used to estimate the quantum yield (QY) of each sample. The calculations assumed identical experimental conditions, including excitation intensity, stripe length, and film preparation protocols; Figure S4. Detailed analysis of ASE characteristics and stripe length dependence for CdTe QDs and CdTe-Ag QD composites encapsulated in SiO_2_ at different molar ratios (1:1 and 1:10). Reference [38] is cited in the Supplementary Materials.

## References

[1] Leanos A.L.A., Cortecchia D., Folpini G., Kandada A.R.S., Petrozza A. (2021). Optical gain of lead halide perovskites measured via the variable stripe length method. Adv. Opt. Mater..

[2] Blood P. (2015). Introduction to optical gain. Quantum Confined Laser Devices: Optical Gain and Recombination in Semiconductors.

[3] Qaid SM H., Ghaithan H.M., Bandar Ali A.A., Aldwayyan A.S. (2021). Achieving optical gain of the CsPbBr_3_ perovskite quantum dots and influence of the variable stripe length method. ACS Omega.

[4] Fox M. (2010). Quantum confinement. Optical Properties of Solids.

[5] Guzelturk B., Kelestemur Y., Olutas M., Delikanli S., Demir H.V. (2014). Amplified Spontaneous Emission and Lasing in Colloidal Nanoplatelets. ACS Nano.

[6] Jung H., Ahn N., Klimov V.I. (2021). Prospects and challenges of colloidal Quantum dot laser diode. Nat. Photonics.

[7] Zhou T., Qiao F., Qian S., Muhammad S., Li H., Liu Y. (2024). CdTe QDs@SiO_2_ composite material for efficient photocatalytic degradation of tetracycline composites. Green Chem. Eng..

[8] Wang J., Wang L., Su x., Gao D., Yu H. (2022). CdTe quantum dot based self-supporting films with enhanced stability for flexible light- emitting devices. Soft Matter.

[9] Sahu A., Kumar D. (2022). Core-shell quantum dots: A review on classification, materials, application, and theoretical modelling. J. Alloys Compd..

[10] Zhou H., Yang C., Liao M., Li M., Diao N., Wu S. (2022). Exploring the mechanism of CdTe quantum dots as fluorescent probe to detect Hg(II) ion from the perspective of fluorescence polarization and light scattering. Chem. Phys. Lett..

[11] Lia Y., Li B.Q. (2014). Use of CdTe quantum dots for high temperature thermal sensing. RSC Adv..

[12] Thuy UT D., Toan P.S., Chi TT K., Khang D.D., Liem N.Q. (2010). CdTe quantum dots for an application in the life sciences. Adv. Nat. Sci. Nanosci. Nanotechnol..

[13] An L.M., Yang Y.Q., Su W.H., Yi J., Liu C.X., Chao K.F., Zeng QH J. (2010). Enhanced fluorescence from CdTe quantum dots self- assembled on the surface of silver nanoparticles. J. Nanosci. Nanotechnol..

[14] Zheng J., Nicovich P.R., Dickson R.M. (2007). Highly fluorescent noble metal quantum dots. Annu. Rev. Phys. Chem..

[15] Bitton O., Gupta S.N., Haran G. (2019). Quantum dot plasmonics: From weak to strong coupling. Nanophotonics.

[16] Di Carlo V., Prete P., Dubrovskii V.G., Berdnikov Y., Lovergine N. (2017). CdTe nanowires by Au-catalyzed metalorganic vapor phase epitaxy. Nano Lett..

[17] Xia Y.S., Cao C., Zhu C.Q. (2008). Two distinct photoluminescence response of CdTe quantum dots to Ag (I). J. Lumin..

[18] Ragab A.E., Gadallah A.-S., Ros T.D., Mohamed M.B., Azzouz I.M. (2013). Luminescence enhancement of CdTe QDs using surface plasmon of Ag NPs. Opt. Commun..

[19] Dutta A., Almutairi A.S., Joseph J.P., Baev A., Petrou A., Zeng H., Prasad P.N. (2022). Exploring magneto-optic properties of colloidal two-dimensional copper-doped CdSe nanoplatelets. Nanophotonics.

[20] Delikanli S., Isik F., Durmusoglu E.G., Erdem O., Shabani F., Canimkurbey B., Kumar S., Baruj H.D., Demir H.V. (2022). Observation of optical gain from aqueous quantum well heterostructures in water. Nanoscale.

[21] Shaklee K.L., Nahory R.E., Leheny R.F. (1973). Optical gain in semiconductors. J. Lumin..

[22] Valenta J., Luterova K., Tomasiunas R., Dohnalova K., Honerlage B., Pelant L. (2003). Optical Gain Measurements with Variable Stripe Length Technique.

[23] Negro L.D., Pavesi L., Pucker G., Franzo G., Priolo F. (2001). Optical gain in silicon crystals. Opt. Mater..

[24] Negro L.D., Bettotti P., Cazzanelli M., Pacifici D., Pavesi L. (2004). Applicability conditions and experimental analysis of the variable stripe length method for gain measurements. Opt. Commun..

[25] Thomas R., Smowton P.M., Blood P. (2015). Radiative recombination rate measurement by the optically pumped variable stripe length method. Opt. Express..

[26] Kobayashi Y., Katakami H., Mine E., Nagao D., Konno M., Marzan L.M.L. (2005). Silica coating of silver nanoparticles using a modified Stober method. J. Colloid Interface Sci..

[27] Dey S., Mishra S.M., Roy A., Roy A., Senapati D., Satpati B. (2023). Multiple gold nanoparticle cores within a single SiO_2_ shell for preservable solid state surface enhanced Raman scattering and catalytic sensing. Appl. Nano Mater..

[28] Yu W.W., Qu L., Guo W., Peng X. (2003). Experimental determination of the extinction coefficient of CdTe, CdSe, and CdS nanocrystals. Chem. Mater..

[29] Koshel D., Barba D., Martin F., Ross G.G. (2009). System for variable stripe length optical gain measurements in structures containing silicon nanocrystals. Proceedings of the Photonics North 2009.

[30] Kim I., Choi G.E., Mei M., Kim M.W., Kim M., Kwon Y.W., Jeong T.I., Kim S., Hong S.W., Kyhm K. (2023). Gain enhancement of perovskite nanosheets by a patterned waveguide: Excitation and temperature dependence of gain saturation. Light Sci. Appl..

[31] Kimura Y., Ito A., Miyachi M., Takahashi H., Watanabe A., Ota H., Ito N., Tanabe T., Sonobe M., Chikuma K. (2001). Optical gain and optical internal loss of GaN-based laser diodes measured by variable stripe length method with laser processing. Jpn. J. Appl. Phys..

[32] Kim B.J., Kyhm K. (2007). Optical modal gain saturation of exciton-exciton scattering and electron-hole plasma in ZnO. J. Korean Phys. Soc..

[33] Sharma R., Kim B., Cho C., Kyhm K. (2009). Modal optical gain and cavity mode analysis of unstructured and optically structured ZnO nanocrystalline thin films. J. Phys. D Appl. Phys..

[34] Kim S., Sharma R., Kim B., Yang H.S., Kyhm K. (2009). Modal gain enhancement by cylindrical waveguide and gain saturation in CdSe nanocrystal quantum dots. J. Phys. D Appl. Phys..

[35] Haridas M., Tripathi L.N., Basu J.K. (2011). Photoluminescence enhancement and quenching in metal semiconductor quantum dot hybrid arrays. Appl. Phys. Lett..

[36] Kulakovich O.S., Korbutyak D.V., Kalytchuk S.M., Budzulyak S.I., Kapush O.A., Trishchuk L.I., Vaschenko S.V., Stankevich V.V., Ramanenka A.A. (2012). Influence of conditions for synthesis of CdTe nanocrystals on their photoluminescence properties and plasmon effects. J. Appl. Spectrosc..

[37] Ragab A.E., Gadallah A.-S., Mohamed M.B., Azzouz I.M. (2014). Photoluminescence and upconversion on Ag/CdTe quantum dots. Opt. Laser Technol..

[38] Wei X., Wang R., Luo Z., Tao P. (2021). One-Step Synthesis of High-Quality CdTe Quantum Dots Using Hydroxylamine Hydrochloride to Reduce Sodium Tellurite. Appl. Phys. A.

