# Supplementary material for: Gain Saturation of Encapsulated CdTe-Ag Quantum Dot Composite in SiO2
Source: Nanomaterials (Basel). 2024 Dec 4;14(23):1950. doi: 10.3390/nano14231950 (PMC11643136; doi:10.3390/nano14231950)
Supplement: Supplementary file 1 [file nanomaterials-14-01950-s001.zip › nanomaterials-3319573-SI.pdf]

Supporting Information

S1. Sample information

Silver perchlorate ($AgClO_4$) (Kanto Chemical Co., Inc., 99%), sodium borohydride (NaBH 4) (Samchun Chemical Co., Ltd., 98%), and trisodium citrate dihydrate (Na-cit) (Neofroxx GmbH., 99%) were used as silver precursor, reducing reagent, and stabilizer for preparation of silver nanoparticles, respectively. Special grade reagents (Sigma-Aldrich.) of tetraethylorthosilicate (TEOS) (98%) and ethanol (99%) were used for silica coating and ammonia (25% aqueous solution) and dimethylamine (DMA) (50%) were used as catalysts for a sol–gel reaction of TEOS. All chemicals were used as received. Ultrapure deionized water was used in all the preparations.

S1-1. Synthesis of Silver Nanoparticles

Silver nanoparticle colloids were prepared by the reduction of silver perchlorate ($AgClO_4$) with sodium borohydride ($NaBH_4$) in the presence of sodium citrate (Na-cit) as a stabilizer, following a modified protocol based on a previous study [1]. In this process, 500 µl of 0.2 M $AgClO_4$ solution was added dropwise to 100 ml of 3 mM $NaBH_4$ and 10 mM Na-cit in an ice-cooled water bath under vigorous stirring. The reaction mixture turned yellow within a few minutes, indicating the formation of silver nanoparticles.

The synthesized Ag nanoparticles exhibited an absorption peak at ~389 nm, consistent with the localized surface plasmon resonance (LSPR) of Ag nanoparticles with an average size of ~10 nm. This size estimation is based on the absorption peak and aligns with the TEM-based size distribution reported in the referenced study. Details of the synthesis protocol and associated characterization can be found in [1].

S1-2. Preparation of CdTe QD and CdTe-Ag QD Composites Encapsulated in SiO_2

CdTe QD and CdTe-Ag QD composites encapsulated in SiO_2 were prepared using a modified Stöber method. For pure CdTe QD encapsulation, 4 mL of CdTe QD solution (0.001 mol L^{-1}), 0.3 mL of ammonia (25% aqueous solution), and 0.3 mL of tetraethyl orthosilicate (TEOS) were added to 10 mL of ethanol under vigorous stirring. The reaction was carried out for 5 hours at room temperature. After the reaction, the suspension was centrifuged at 15,000 rpm for 20 minutes, washed three times with distilled water, and finally redispersed in 10 mL of distilled water to obtain the CdTe/SiO_2 solution.

For CdTe-Ag QD composites, Ag QDs were mixed with CdTe QDs in two different molar ratios (1:1 and 1:10) before the encapsulation process. The mixed solutions were subjected to the same encapsulation procedure as described above. This yielded CdTe-Ag QD/SiO_2 composites with different Ag QD concentrations.

S1-3. Encapsulation of Multiple Nanoparticles within SiO_2 Shells

As shown in previous TEM studies [1, 2, 3], higher concentrations of nanoparticles often result in multiple cores being encapsulated within a single SiO_2 shell. This supports our hypothesis regarding the internal structure of our composite system.

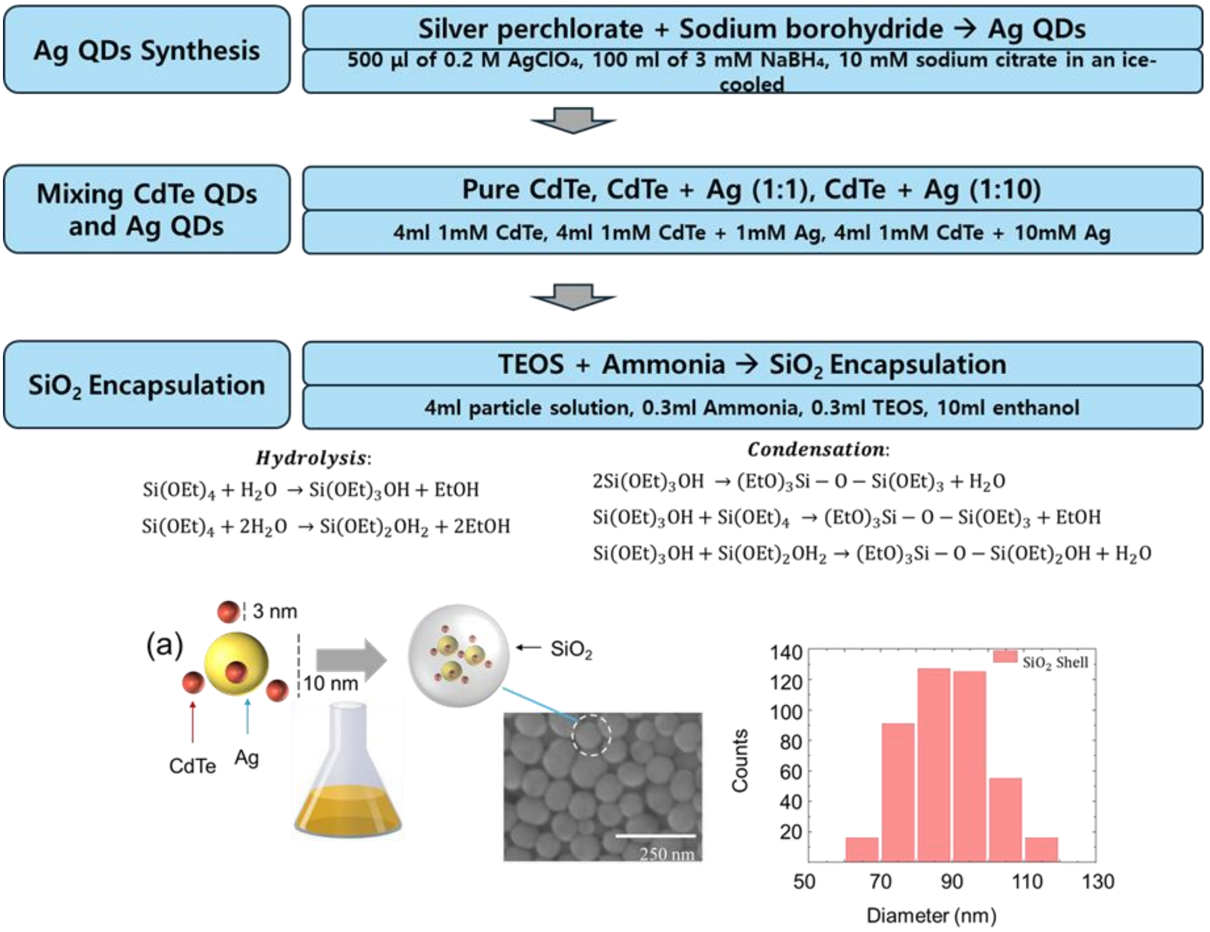

Figure S 1. Stepwise synthesis process of CdTe/Ag QD composites encapsulated in SiO_2.
(1) Silver nanoparticles (Ag QDs) were synthesized via chemical reduction.
(2) CdTe QDs were mixed with Ag QDs at molar ratios of 1:1 and 1:10.
(3) The resulting mixture was encapsulated in silica using a modified Stöber method.
The SEM image shows uniform SiO_2 shells, and the histogram represents the shell thickness distribution, confirming the consistency of the encapsulation process.

S2. Absorbance Spectra in Logarithmic Scale

The absorbance spectra of CdTe QDs, Ag QDs, and their composites were replotted on a logarithmic scale to better highlight subtle spectral features. The shoulder near 425 nm, characteristic of CdTe QDs, is more distinct in the logarithmic representation, further supporting the attribution of this feature to CdTe QDs. This visualization also emphasizes the overlap between the CdTe QDs' spectral shoulder and the broader absorbance band of Ag QDs, indicating plasmonic interactions.

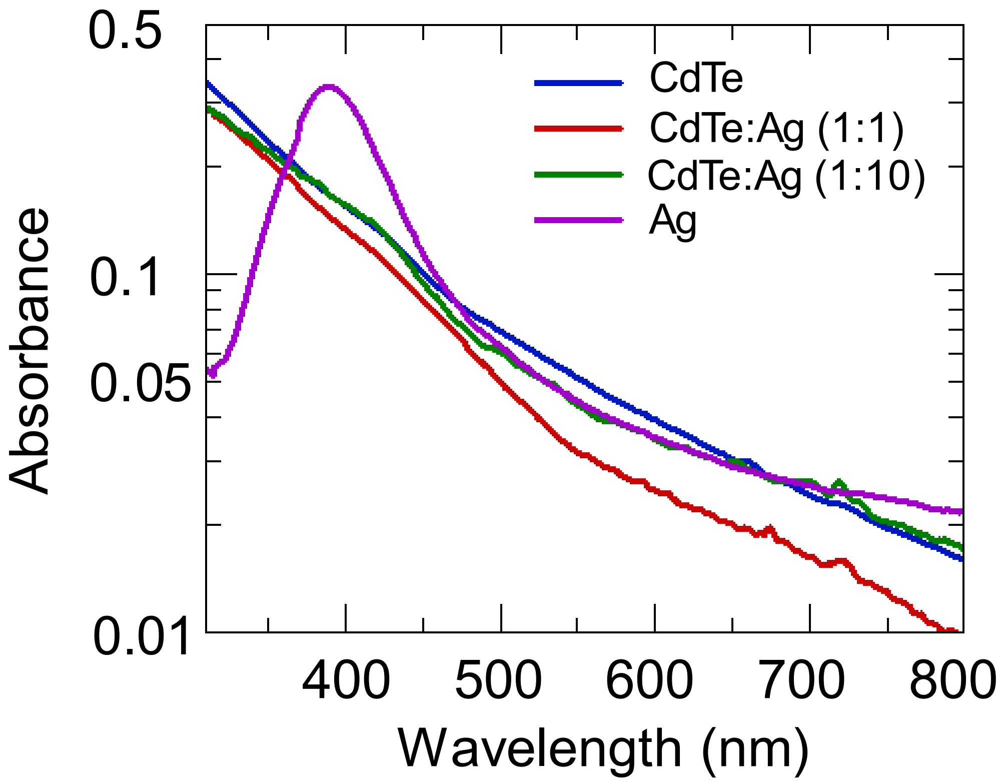

Figure S 2. Absorption spectra of CdTe QDs, Ag QDs, and CdTe-Ag composites at molar ratios of 1:1 and 1:10. The absorption peak of CdTe QDs at ~425 nm and the localized surface plasmon resonance (LSPR) peak of Ag QDs at ~389 nm are visible. The composite spectra show slight shifts and broadenings, which are attributed to plasmonic interactions between CdTe QDs and Ag QDs. Data were measured in solution after particle recovery, assuming consistent concentration across all samples.

S3. **Relative Quantum Yield Estimation**

To estimate the relative quantum yields (QY) of the CdTe-Ag composites, the ASE spectra of the samples were integrated over the emission wavelength range. Figure S4 shows the ASE spectra of pure CdTe QDs, CdTe-Ag (1:1), and CdTe-Ag (1:10) composites, with the corresponding integrated areas representing the relative ASE intensities[4].

The calculated QY values, normalized to the QY of the pure CdTe QDs (10%, as reported by the manufacturer), are as follows:

- CdTe QDs: 10% (reference)
- CdTe-Ag (1:1): 8.73%
- CdTe-Ag (1:10): 11.8%

These results suggest that the quantum yield remains relatively consistent across the samples, with minor variations likely due to experimental uncertainties

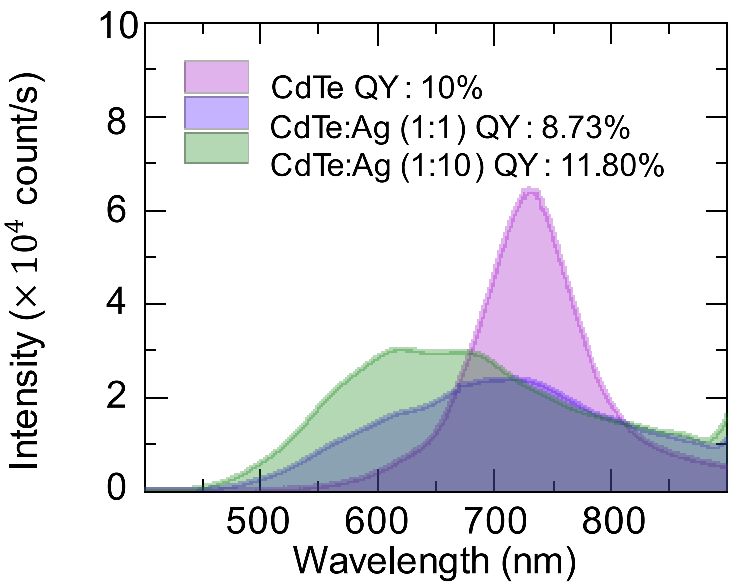

Figure S 3. Integrated ASE spectra of CdTe QDs and CdTe-Ag composites with different Ag QD molar ratios (1:1 and 1:10). The areas under the curves represent the relative ASE intensities, which were used to estimate the quantum yield (QY) of each sample. The calculations assumed identical experimental conditions, including excitation intensity, stripe length, and film preparation protocols.

S4. Determination of x_{th} and x_{sat}

The threshold stripe length (x_{th}) and saturation stripe length (x_{sat}) were determined from the ASE intensity data as a function of stripe length for different emission wavelengths. The specific methods are as follows:

1. **Threshold Stripe Length (x_{th})**:

   To ensure an objective determination of the threshold stripe length (x_{th}), we analyzed the ASE intensity (I_{ASE}) as a function of stripe length (x) in a logarithmic scale. The procedure is as follows

   - Logarithmic Transformation : The ASE intensity data (I_{ASE}) were transformed to $\ln(I_{ASE})$ vs x, resulting in a plot where the data in the linear regime (amplification regime) form a straight line.
   - Fitting in the Linear Regime : The linear regime was identified by selecting the initial range of stripe lengths where $\ln(I_{ASE})$ increased approximately linearly. A linear fit ($y = ax + b$) was applied to this region.
   - Extrapolation to x_{th} : The intercept of the fitted line with the x-axis ($x_{th} = -\frac{b}{a}$) was calculated, providing a mechanical and consistent definition of the threshold stripe length.

2. **Saturation Stripe Length (x_{sat})**:

   - x_{sat} corresponds to the stripe length at which the ASE intensity reaches a plateau, indicating saturation of gain due to the depletion of excited carriers.
   - x_{sat} was determined as the point where the ASE intensity slope ($\frac{dI^2}{d^2x}$) approached zero within experimental noise limits.

3. **Calculation of $\boldsymbol{L_{amp}}$**:

   - The amplification range ($\boldsymbol{L_{amp}}$) is calculated $\boldsymbol{L_{amp}} = x_{sat} - x_{th}$. This represents the effective stripe length over which optical gain is observed.

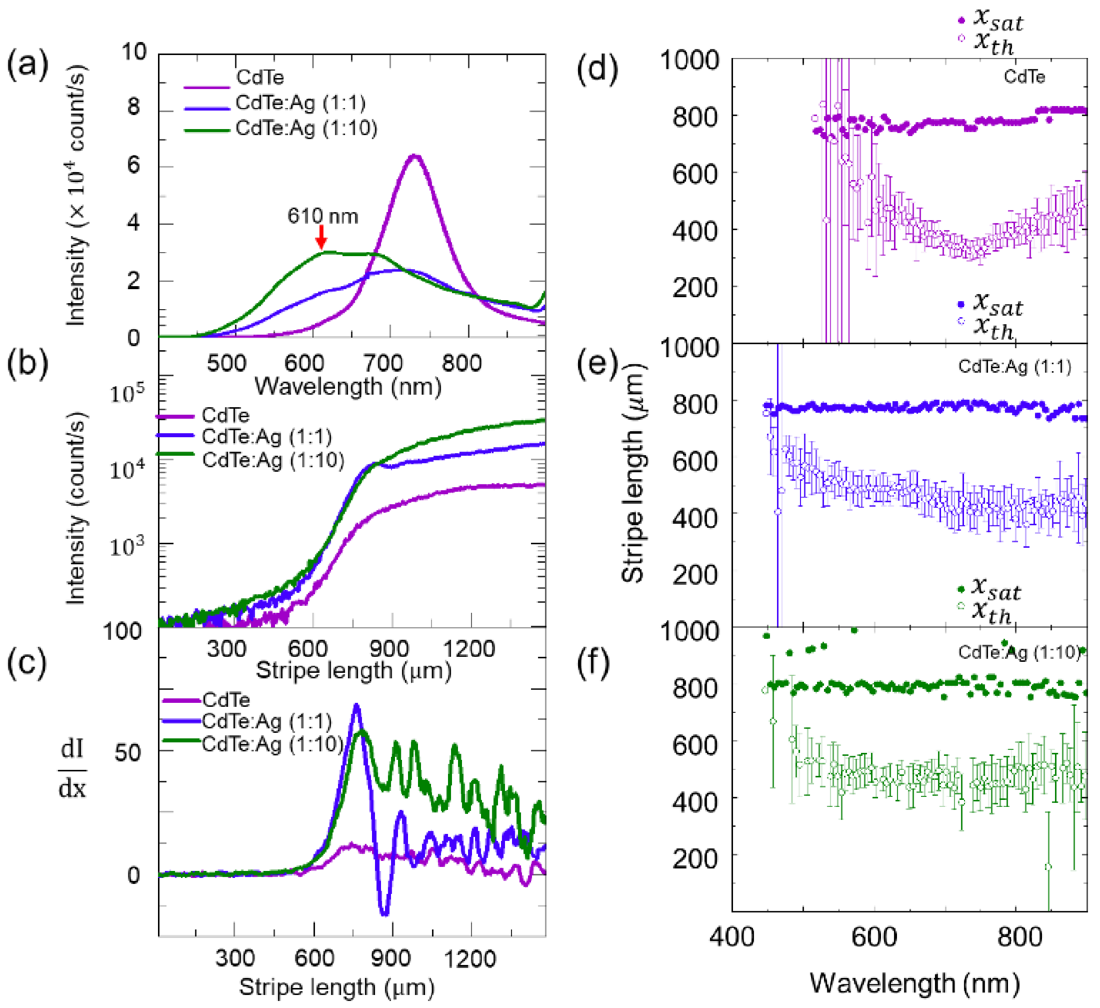

Figure S 4. Detailed analysis of ASE characteristics and stripe length dependence for CdTe QDs and CdTe-Ag QD composites encapsulated in SiO_2 at different molar ratios (1:1 and 1:10).

(a) ASE spectra for CdTe QDs and CdTe-Ag QD composites under identical stripe length conditions. The red arrow highlights the 610 nm wavelength, which is further analyzed in (b) and (c).

(b) ASE intensity at 610 nm as a function of stripe length, demonstrating that samples with Ag QDs exhibit higher ASE intensities compared to pure CdTe QDs, indicating plasmonic enhancement.

(c) Differential analysis of ASE intensity at 610 nm as a function of stripe length. The peak values are larger for samples containing Ag QDs, further supporting the plasmonic enhancement effect.

(d) Wavelength-dependent threshold (x_{th}) and saturation (x_{sat}) stripe lengths for pure CdTe QDs encapsulated in SiO_2.

(e) Similar analysis for CdTe-Ag QD composites with a 1:1 molar ratio, showing shifts in x_{th} and x_{sat} due to plasmonic interactions.

(f) Analysis for CdTe-Ag QD composites with a 1:10 molar ratio, where higher Ag concentrations further modulate x_{th} and x_{sat} values.

Reference

1. Kobayashi Y.; Katakami H.; Mine E.; Nagao D.; Konno M.; Marzan L. M. L. Silica coating of silver nanoparticles using a modified Stob¨er method. J. Colloid Interface Sci. 2005, 283, 392-396. https://doi.org/10.1016/j.jcis.2004.08.184
2. Wang J.; Wang L.; Su X.; Gao D.; Yu H. CdTe Quantum Dot-Based Self-Supporting Films with Enhanced Stability for Flexible Light-Emitting Devices. Soft Matter 2022, 18, 7324-7332. https://doi.org/10.1039/D2SM01108E
3. Dey S.; Mishra S. M.; Roy A.; Roy A.; Senapati D.; Satpati B. Multiple gold nanoparticle cores within a single SiO2 shell for preservable solid state surface enhanced Raman scattering and catalytic sensing. Appl. Nano Mater. 2023, 6, 15606−15619. https://doi.org/10.1021/acsanm.3c02455
4. Wei, X.; Wang, R.; Luo, Z.; Tao, P. One-Step Synthesis of High-Quality CdTe Quantum Dots Using Hydroxylamine Hydrochloride to Reduce Sodium Tellurite. Appl. Phys. A 2021, 127, 1–5. https://doi.org/10.1007/s00339-021-05138-9.
